# The Src family kinase LCK cooperates with oncogenic FLT3/ITD in cellular transformation

**DOI:** 10.1038/s41598-017-14033-4

**Published:** 2017-10-23

**Authors:** Alissa Marhäll, Julhash U. Kazi, Lars Rönnstrand

**Affiliations:** 1 Division of Translational Cancer Research, Department of Laboratory Medicine, Lund University, Medicon Village, Lund, Sweden; 2 Lund Stem Cell Center, Department of Laboratory Medicine, Lund University, Lund, Sweden; 3 Division of Oncology, Skåne University Hospital, Lund, Sweden

## Abstract

The non-receptor tyrosine kinase LCK belongs to the SRC family of kinases. SRC family kinases are proto-oncogenes that have long been known to play key roles in cell proliferation, motility, morphology and survival. Here we show that LCK regulates the function of the type III receptor tyrosine kinase FLT3 in murine pro-B cells. We observed that expression of LCK significantly enhances the colony forming capacity of the constitutively active FLT3 mutant FLT3-ITD (internal tandem duplication). Furthermore, cells expressing LCK developed tumor earlier compared to cells transfected with empty control vector. Staining of the tissues from mouse xenografts showed higher Ki67 staining in cells expressing LCK suggesting that expression of LCK enhances the FLT3-ITD-mediated proliferative capacity. LCK expression did not affect either FLT3-WT or FLT3-ITD -induced AKT, ERK1/2 or p38 phosphorylation. However, LCK expression significantly enhanced FLT3-ITD-mediated STAT5 phosphorylation. Taken together, our data suggest that LCK cooperates with oncogenic FLT3-ITD in cellular transformation.

## Introduction

Oncogenic mutations or overexpression of tyrosine kinases are very common in a wide range of cancers. Several members of type III receptor tyrosine kinases including FLT3, KIT and CSF1R have been implicated in hematopoietic malignancies^1,2^. FLT3 was found to be mutated in as high as 35% of acute myeloid leukemia (AML) and in a small portion of acute lymphoblastic leukemia (ALL)^3,4^. One of the most common FLT3 mutations includes the internal tandem duplication (ITD) in the juxtamembrane domain of the receptor. Although the wild-type receptor needs its ligand, FLT3 ligand (FL), to trigger downstream signaling, FLT3-ITD is constitutively active and can activate downstream signaling cascade in the absence of ligand stimulation. The downstream signaling is tightly controlled by associating proteins, which directly or indirectly interact with the activated receptor. Associating proteins include protein kinases, protein phosphatases, ubiquitin ligases and adaptor proteins^5–12^. Protein kinase, such as SYK^6^ and FYN^13^, cooperate with oncogenic FLT3-ITD, while CSK^14^ and ABL2^15^ partially block mitogenic signaling. The protein tyrosine phosphatase DEP1 negatively regulates FLT3-ITD-mediated colony formation^16^ and loss of STS1/STS2 function results in hyperactivation of FLT3^11^. In contrast, association of another phosphatase, SHP2, seems to be essential for FLT3-ITD-mediated cellular transformation^17^. These findings suggest that the role of protein kinases or phosphatases cannot be simplified and specific kinase or phosphatase can act as negative or positive regulators of FLT3 signaling. Furthermore, although several E3 ubiquitin ligases such as SOCS2^18^, SOCS6^19^, SLAP^20^ and SLAP2^9^ accelerate ubiquitination-directed degradation of FLT3, signaling molecules play diverse roles in regulating mitogenic signaling. For instance, SLAP depletion partially blocked activation of FLT3 downstream signaling cascades^20^ while depletion of SOCS6 accelerated mitogenesis^19^. Therefore, knowledge of individual FLT3 interacting proteins is required in order to understand how FLT3 downstream signaling is regulated. The lymphocyte-specific protein tyrosine kinase, LCK, is a member of the SRC family of kinases (SFKs). SFKs are a family of 11 non-receptor tyrosine kinases^21^. LCK has important functions in T cell development, homeostasis and activation^22^. LCK knockout mice display a strong decline in the CD4 and CD8 positive thymocyte population and carry only a few peripheral T cells^23^. Although LCK under normal physiological conditions primarily is expressed in T cells and in some subpopulations of B cells^24^, it is highly expressed both in B and T cell leukemia^25,26^ and contributes to the malignant phenotype. Loss of LCK expression in T-cell leukemia cells, or peripheral T lymphocytes, results in impaired T cell receptor activation^27,28^. In B-cell leukemia, cells with hyperphosphorylated FLT3 also display high levels of LCK phosphorylation^29^ suggesting a possible role of FLT3 in LCK activation or *vice versa*.

Apart from cells of hematopoietic origin, LCK is also aberrantly expressed in a number of other cancer types, including breast cancer, colon cancer and small cell lung carcinoma^30–32^ suggesting that it has general cancer promoting activities. Several studies have reported high levels of LCK expression in acute myeloid leukemia. Early studies indicated high expression of LCK in leukemic cells from patients with less differentiated AML, i. e. AML-0 and AML-1^33^. However, a role of LCK in FLT3-dependent AML has not yet been defined. In this report we show, using the proB cell line Ba/F3 that lacks endogenous LCK, that LCK expression is not essential for wild-type FLT3 signaling but plays an important role in oncogenic FLT3-ITD-mediated cellular transformation.

## Results

### LCK expression is dispensable for FLT3-ITD dependent cell viability and survival

**Figure 1:**
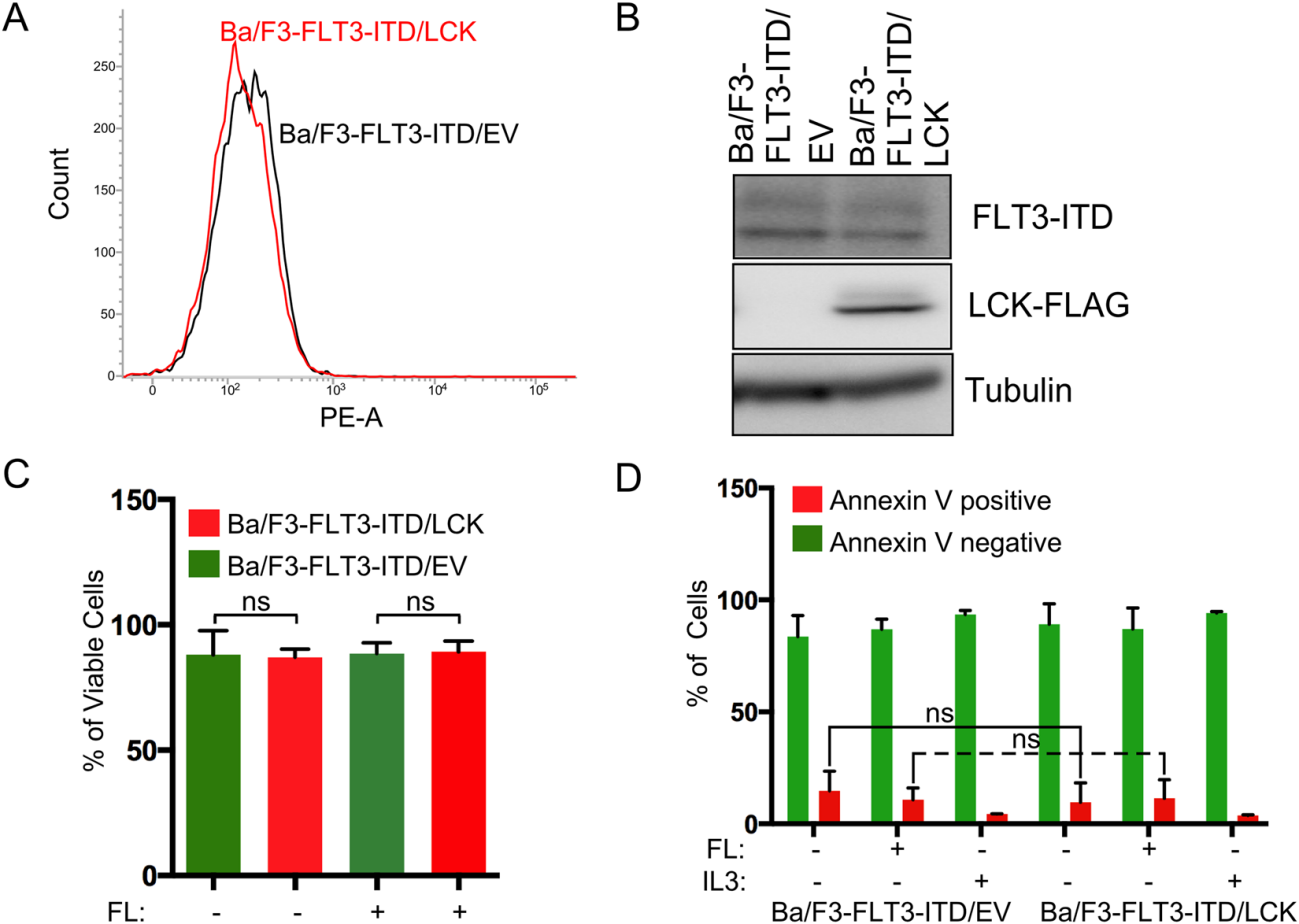
LCK expression does neither alter FLT3-ITD-mediated cell viability nor apoptosis. (**A**) Ba/F3-FLT3-ITD cells expressing LCK, or empty vector (EV), were labeled with phycoerythrin-conjugated anti-FLT3 for analysis with flow cytometry. (**B**) Ba/F3-FLT3-ITD transfected with LCK or empty vector were subjected to Western blot analysis for analysis of total expression of FLT3. Three independent experiments were quantified. The blots were cropped to focus upon the specific proteins indicated. (**C**) Cell viability was assessed by adding Prestoblue reagent 48 h post seeding. The graph represents relative cell viability with or without FL stimulation. (**D**) Cells were washed to remove IL3 and seeded in a 12-well plate in IL3 free medium. After 48 h cells were stained with phycoerythrin (PE)-labeled annexin V and 7-aminoactinomycin D (7-AAD). Cells were then analyzed using flow cytometry. Cells positive for annexin V and 7-aminoactinomycin D (7-AAD), or only for annexin V, were counted as apoptotic cells. Not significant, ns.

It is long been known that SRC family kinases (SFKs) play important roles in mitogenic signaling. SFKs act as an intermediate mediator of various receptor tyrosine kinases. We have shown that SRC^34,35^ and FYN^13^ bind to FLT3 and cooperate with FLT3-ITD in cellular transformation. To understand the role of another SFK, LCK, we generated Ba/F3 cells stably expressing FLT3-ITD and either empty vector or LCK. FLT3 surface expression was analyzed by flow cytometry (Fig. 1A) and the total protein expression was measured by Western blotting (Fig. 1B). FLT3 surface and total expression appeared to be the same for both LCK and empty vector expressing cells, making it a suitable model for studying the impact of LCK on FLT3-ITD mediated biological events. To investigate whether LCK has any effect on cell growth, we examined the cell viability using PrestoBlue assay. Forty-eight hours post seeding of cells we observed, in comparison to the control, no alteration caused by LCK expression on the number of viable cells, regardless of FLT3 ligand stimulation (Fig. 1C). Furthermore, LCK expression neither increased nor decreased the fraction of apoptotic cells in an annexin V/7-AAD assay (Fig. 1D). Thus, we suggest that LCK expression is not essential for FLT3-ITD induced cell viability or survival *in vitro*.

### LCK expression cooperates with FLT3-ITD in colony formation and tumor formation

**Figure 2:**
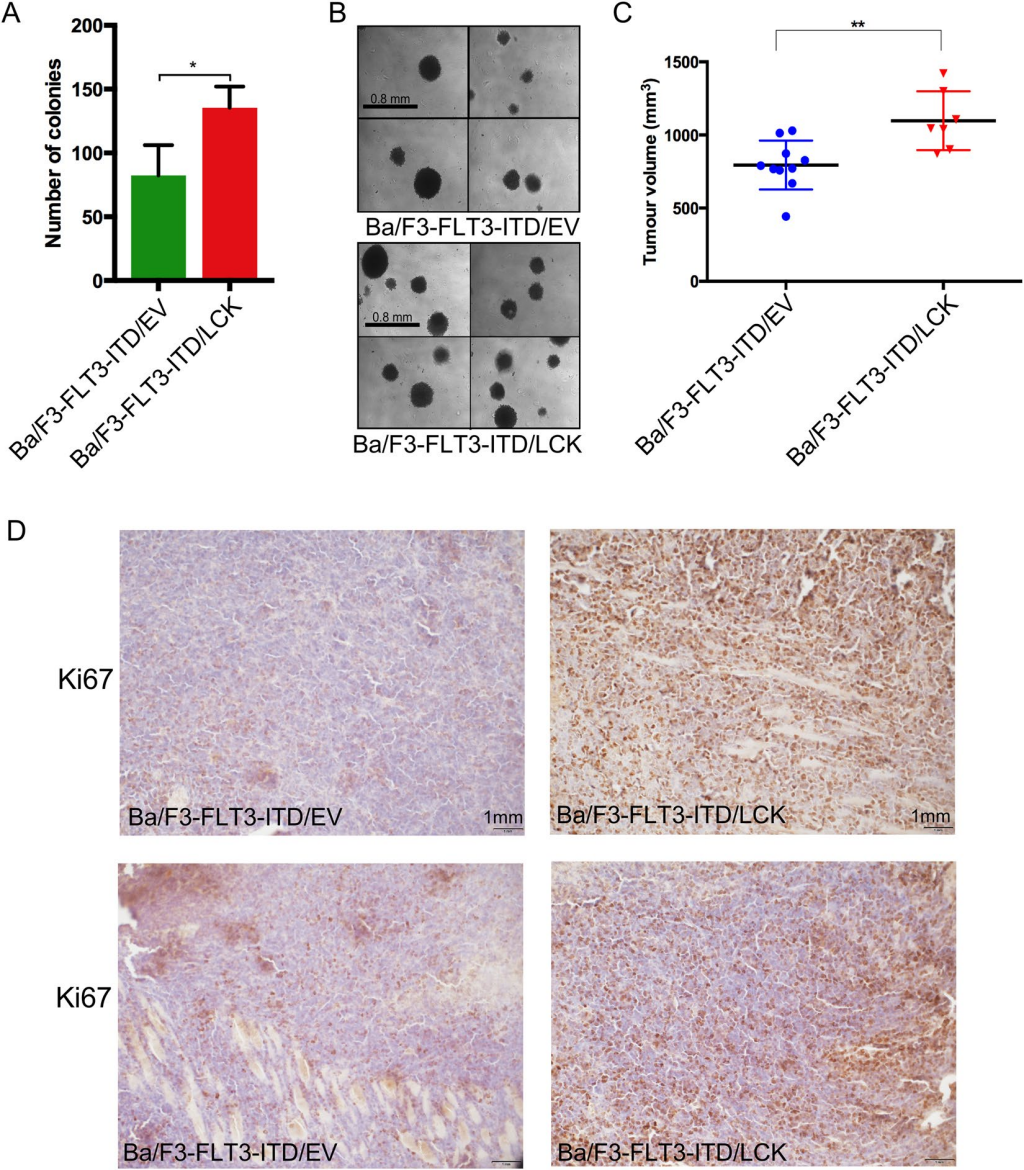
LCK expression promotes colony formation *in vitro* and tumor growth *in vivo*. (**A**) Cells were washed to remove IL-3 and serum. Cells were mixed with 80% methylcellulose medium and seeded in a 24-well plate. Quantified number of colonies formed *p < 0.05. (**B**) For quantification at least 10 pictures were taken per each well with colonies. (**C**) NOD/SCID mice were injected subcutaneously with 1 × 10^6^ cells. The total volume size was measured after mice were sacrificed. (**D**) IHC of the tumor paraffin sections stained for Ki67, the photos are taken at 20X magnification. Empty vector, EV. *p < 0.05, **p < 0.01.

Since we did not see any effect of LCK on FLT3-ITD-mediated *in vitro* cell survival, we asked whether it affects FLT3-ITD-induced *in vitro* colony formation. We observed that the potential to form colonies in the semi-solid medium was significantly increased in cells expressing LCK when compared to cells expressing empty vector control (Fig. 2A). However, the size of the colonies remained basically unchanged compared to controls (Fig. 2B). This suggests that LCK might play a role in FLT3-ITD-mediated cellular transformation. To further verify the *in vitro* findings, NOD/SCID mice were injected subcutaneously with Ba/F3-FLT3-ITD cells transfected with LCK or empty vector. After 25 days mice were sacrificed and the total volume of the tumors was measured. We could show that LCK expression significantly increased the tumor size in xenografted mice (Fig. 2C). To investigate whether the increased tumor size of LCK mice was due to an increase in proliferation, we stained tumor tissues for Ki67 and observed that tumors expressing LCK showed higher Ki67 staining, indicative of a higher proliferative potential (Fig. 2D). Therefore, we suggest that LCK accelerates the FLT3-ITD-mediated transformation potential *in vivo*.

### LCK expression increases FLT3-ITD-mediated STAT5 phosphorylation

**Figure 3:**
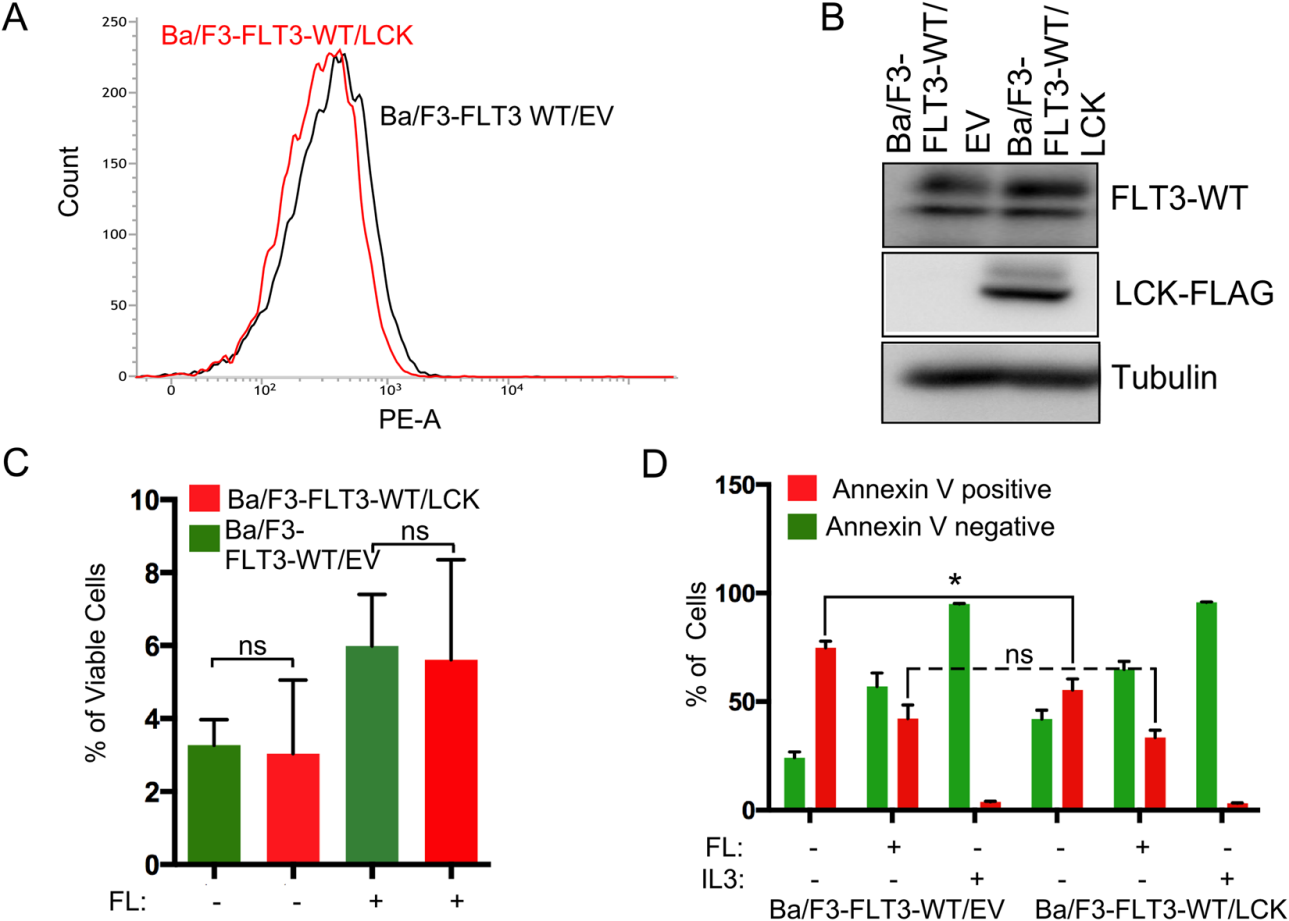
Expression of LCK does not affect wild-type FLT3 mediated cell viability or apoptosis. (**A**) Ba/F3 expressing FLT3 and LCK or empty vector (EV) were labeled with phycoerythrin-conjugated anti-FLT3 and then analyzed by flow cytometry. (**B**) The total amount of FLT3 expression was measured by Western blot. The blots were cropped to focus upon the specific proteins indicated. (**C**) Cells were washed to remove IL3 and seeded in a 96-well plate. PrestoBlue cell viability assay was used to measure viable cells. (**D**) Cells were washed to remove IL3 and seeded in a 12-well plate in IL3-free medium. After 48 h cells were stained with phycoerythrin (PE)-labeled annexin V and 7-aminoactinomycin D (7-AAD). Cells were then analyzed using flow cytometry. Cells positive for annexin V and 7-aminoactinomycin D (7-AAD), or only for annexin V, were counted as apoptotic cells. Not significant, ns; *p < 0.05.

**Figure 4:**
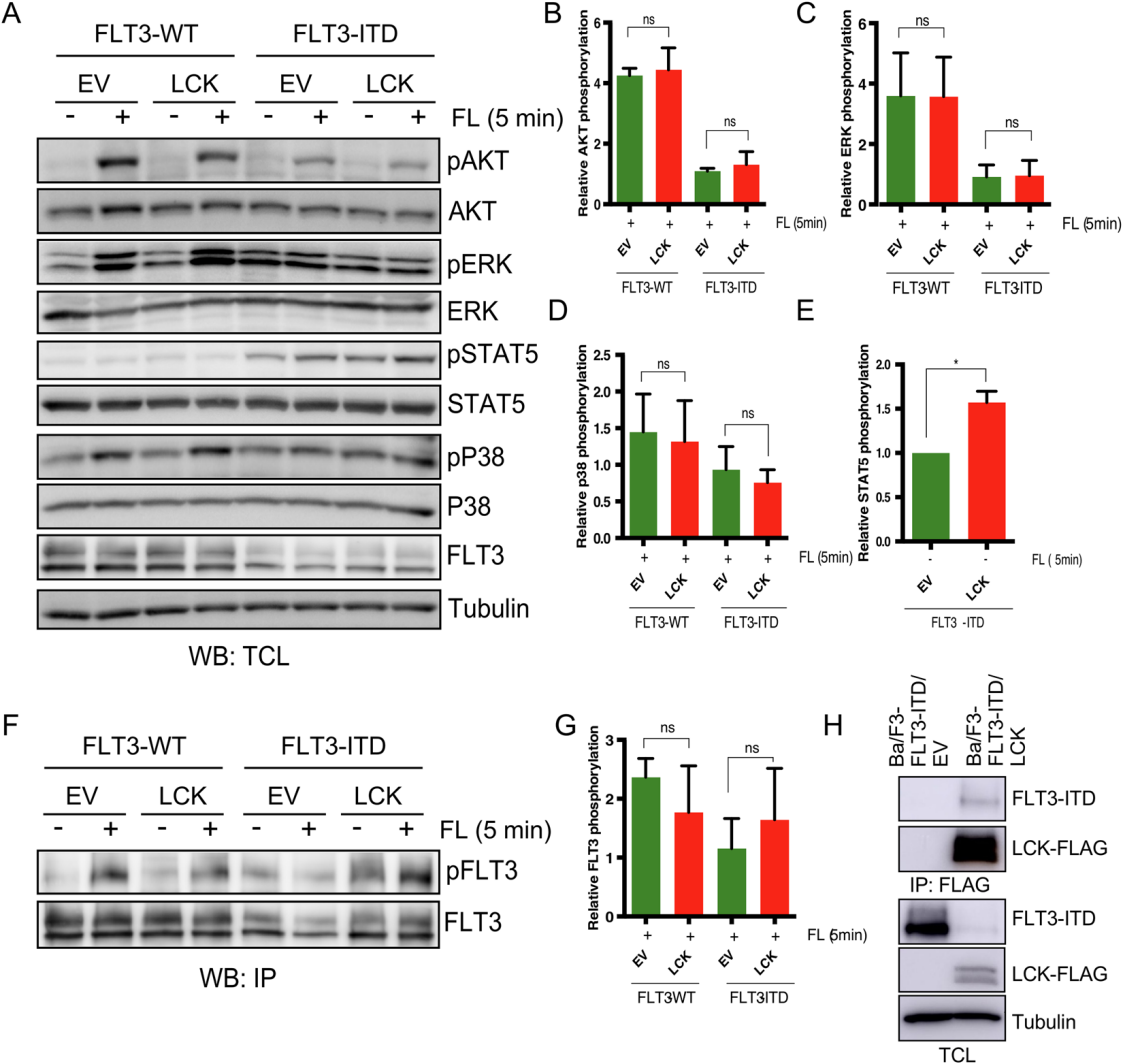
Ba/F3 FLT3-ITD cells expressing LCK display increased STAT5 phosphorylation. (**A**) Ba/F3 cells expressing either wild-type FLT3 or FLT3-ITD and either LCK or empty vector (EV) were washed to remove IL-3 and starved four hours before FL stimulation. Total cell lysates were subjected to Western blotting analysis using phosphospecific antibodies against AKT, ERK1/2, p38 and STAT5. (**B**–**E**) Blots from three independent experiments were quantified. Signals of phosphorylated proteins were normalized against total protein. (**F**) A fraction of the lysate was used for immunoprecipitation using an anti-FLT3 antibody. The blots were cropped to focus upon the specific proteins indicated. (**G**) Blots from three independent experiments from experiment F were quantified. Signals of phosphorylated FLT3 were normalized against total FLT3. (**H**) COS-1 cells were transfected with FLT3-ITD and LCK-FLAG or empty vector. Cells were lysed and lysates were subjected to anti-FLAG antibody immunoprecipitation. Not significant, ns; *p < 0.05.

In contrast to the constitutively active oncogenic mutant FLT3-ITD, wild-type FLT3 is dependent on FL stimulation for activation of the PI3K/AKT, RAS/ERK and p38 pathways^36^. In order to study how LCK regulates downstream signaling of FLT3, we therefore generated Ba/F3 cell lines expressing wild-type FLT3 with LCK or empty vector. Similar to the FLT3-ITD experiments, we used flow cytometry and Western blotting to verify equal surface expression (Fig. 3A) and total expression (Fig. 3B) of wild-type FLT3 in cells expressing LCK or empty vector. Ba/F3 cell line expressing wild-type FLT3 requires IL-3 for proliferation and survival, and LCK does not rescue that phenotype (Fig. 3C). LCK also does not influence the apoptotic rate of Ba/F3 cells in response to FL stimulation, while unstimulated cells expressing LCK displayed significant lower level of apoptotic cells (Fig. 3D). To gain insight into the mediators involved in downstream signaling, we looked at the known FLT3 downstream signaling pathways (Fig. 4A). We observed no change in phosphorylation of AKT (Fig. 4B), ERK (Fig. 4C) or p38 (Fig. 4D). However, STAT5 phosphorylation was significantly increased in Ba/F3 FLT3-ITD expressing LCK compared to the empty vector control (Fig. 4E), explaining the increased proliferation rate of the LCK tumors. However, we did not see any change in total FLT3 tyrosine phosphorylation in the absence or presence of LCK expression (Fig. 4F,G). Finally, we showed that FLT3-ITD was associated with LCK (Fig. 4H).

## Discussion

Receptor tyrosine kinases transduce signals predominantly through interacting proteins. Therefore, understanding of the function of individual interacting partners of a specific receptor is important for understanding the regulation of downstream signaling cascades. Using an SH2 domain array we have recently shown that several SH2 domain-containing proteins such as ABL2, CRK, FYN, ABL1, RASA1, CRKL, LCK, SOCS6, BLK, TNS1, BRK etc. associate with several phosphotyrosine residues in FLT3^15^. We and others have characterized several of those proteins and demonstrated important roles in FLT3 signaling. The role of SRC family kinases, including SRC, LYN, FYN and HCK, has been outlined. Activation of FLT3 results in elevated tyrosine phosphorylation of LYN and SRC, and inhibitors targeting the SRC family kinases significantly reduced cell viability in FLT3-ITD-dependent AML cell lines, suggesting that the function of SRC family kinases is required for FLT3-induced cell survival^37,38^. While SRC and LYN displayed a role in cell survival, FYN appeared to be involved in FLT3-ITD-mediated cell transformation^13^. A recent report suggests that HCK is involved in FLT3-ITD mediated CDK6 expression and thereby supports cell survival and transformation^39^. Therefore, it is likely that, despite the high structural similarity between SRC family kinases, individual members play distinct roles in FLT3 downstream signaling. In this report, we define the role of LCK in FLT3 signaling.

The function of LCK has mainly been studied in lymphocytes due to the abundant expression in the lymphoid lineage, in particular in T cells. It is highly expressed in several chronic lymphocytic leukemia’s of both B cell and T cell lineages^28,29^. In addition, the myeloid cell line 32D and several non-lymphoid human tumor cell lines also show LCK expression^30,31,34^. Thus, LCK function may not only be restricted to the lymphoid lineage. Several studies have demonstrated a role of LCK in acute myeloid leukemia. Early studies^33^ demonstrated higher expression of LCK in less differentiated cases of AML. In a recent proteomics study the role of individual kinases in AML was investigated and a correlation between high expression of LCK correlated with good response to a PI3K/mTOR-specific inhibitor^40^. Using a bioinformatics approach aiming at identifying relevant therapeutic targets in AML^41^, several transcripts were identified that were differentially expressed between normal bone marrow samples and AML samples. Based on these data, they constructed a protein-protein interaction network and identified, among other proteins, LCK as one of the proteins of the hub nodes. Additionally, activation of FLT3 in AML samples resulted in abundant phosphorylation in the activation loop of LCK^29^.

Taken together, these data collectively suggest that LCK might play a role in FLT3-ITD-mediated AML. Using Ba/F3 cells, lacking endogenous LCK expression, as a model system, we could show that LCK expression did not contribute to overall tyrosine phosphorylation of FLT3 suggesting that LCK does not have a role in the FLT3 activation process. Furthermore, LCK did not contribute to FLT3-ITD-induced *in vitro* cell viability, but enhanced colony formation capacity, suggesting that LCK regulates distinct signaling pathway downstream of FLT3. This is also supported by the data that STAT5 phosphorylation, but not AKT, ERK1/2 and p38 phosphorylation, was enhanced in the presence of LCK. This is similar to what has been described for PCP-ALL cells, where a PAX5 fusion protein drives overexpression of LCK. In those cells, there is an LCK-dependent hyperphosphorylation of STAT5^42^. Similar to *in vitro* colony formation data, mice injected with cells expressing LCK and FLT3-ITD developed tumors quicker than cells lacking LCK expression. Collectively, our data suggest that LCK enhances the FLT3-ITD mediated transformation potential by cooperating with STAT5 pathway activation. Thus, LCK is a potential target for the development of selective SRC family kinase inhibitors that could potentially be used together with FLT3 inhibitors to treat patients with FLT3-ITD positive AML.

## Materials and Methods

### Cell culture and transfection

Murine pro-B cell line Ba/F3 (DSMZ, Braunschweig, Germany), was cultured in RPMI-1640 medium (Hyclone, Thermo Scientific, Waltham, MA) supplemented with 10% heat-inactivated fetal bovine serum (Life Technologies, Carlsbad, CA), 10 ng/ml recombinant murine interleukin 3 (IL3) and 100 units/ml penicillin, and 100 μg/ml streptomycin. Generation of Ba/F3-FLT3-ITD cells was described previously^1^. FLT3-ITD-transfected Ba/F3 cells were then further transfected with the pMSCV-FLAG-LCK or empty vector construct. Cells were selected with 0.8 mg/ml G-418 for 2 weeks. Transfected cells were maintained in Ba/F3 medium as previously described^43^. Cells were grown at 37 °C in a humidified atmosphere containing 5% CO_2_.

### Immunoprecipitation and Western blotting

For signaling studies, before stimulation, Ba/F3 cells were starved for 4 hours in RPMI-1640 medium without serum or cytokines. Cells were stimulated with 100 ng/mL FL (ORF Genetics, Kópavogur, Iceland) for the indicated periods of time at 37 °C. Cells were washed once with cold PBS and lysed in lysis buffer [40 mM Tris–HCl (pH 8.0), 120 mM NaCl, 0.1% Nonidet-P40] supplemented with protease inhibitors. Lysates were cleared by centrifugation at 14,000 × g for 10 min at 4 °C. For immunoprecipitation, 1 µg of antibody was used for 1 ml of cell lysate. Lysate and antibodies were mixed and kept on ice for 1 h before adding 20 µl of Dynabeads Protein G (ThermoFisher Scientific) followed by mixing end-over-end for 20 minutes. Beads were then washed three times with lysis buffer. Where the total cell lysates were used, equal amounts of proteins were electrophoretically separated on 8% SDS–PAGE gel and transferred to a PVDF membrane (Amersham, Arlington Heights, IL). Membranes were blocked with 5% non-fat dry milk in PBS-T, and probed with antibodies towards FLT3 (1 µg/ml, homemade, previously described^44,45^), 4G10 (1 µg/ml, Millipore), phospho-p38 (1 µg/ml, BD Biosciences) and p38 (1 µg/ml, BD Biosciences), phospho-ERK1/2, ERK2, phospho-STAT5, STAT5 and AKT (all at the dilution 1:200 Santa-Cruz Biotechnology), phospho-AKT (1:500, Epitomics), FLAG(1:2000, Sigma-Aldrich), LCK (1:200, Santa Cruz) and β-actin (1:5000, Sigma–Aldrich), followed by incubation with a horseradish peroxidase-labeled secondary antibody (1:5000). Immunodetection was performed by using ECL (Millipore Corporation, Billerica, MA) and a CCD camera (LAS-3000, Fujifilm, Tokyo, Japan). Signal intensity was quantified by MultiGauge software (Fujifilm).

### Cell proliferation, apoptosis, and colony formation assay

Cells were washed three times to remove cytokine before all experiments. annexin V and 7-aminoactinomycin D (7-AAD) apoptosis kit (BD Biosciences) was used to measure apoptosis in cytokine-depleted cells. Cells positive for annexin V and both annexin V/7-AAD were counted as apoptotic cells. To measure cell proliferation, 10,000 cells were seeded into each well of a 96-well plate and incubated for 48 h. PrestoBlue (Thermo Fisher Scientific) was used to measure cell viability. Semi-solid methylcellulose medium (Stem Cell Technologies) was used for colony formation assay. Around 500 cells were seeded and cultured for seven days before counting colonies.

### Animal work

NOD/SCID female mice were purchased from Charles River laboratories. 1 × 10^6^ control or LCK expressing Ba/F3 FLT3-ITD cells were injected subcutaneously into 7 mice in each group. The tumor progression was monitored for 25 days. On the day of sacrifice, the tumors were measured and stored in 4% PFA for 24 h followed by the standard protocol of fixation. The experiment was performed under ethical permit from the Swedish Animal Welfare Authority following approved guideline.

### Immunohistochemistry

After formalin fixation, tumors were embedded in paraffin. Tumor sections (4 μm thick) were deparaffinized using xylene, followed by graded ethanol series. Heat-mediated antigen retrieval was performed in retrieval buffer, PT module buffer pH 6 (TA-050-Pm1X), using pressure boiler. Ki67 (1:100, Abcam) antibody staining was performed in Autostainer 480 (Thermo Fisher Scientific Anatomical Pathology, Astmoor Runcorn, UK) for 30 min at room temperature, washed (x2) and incubated with secondary antibody for 30 min. Developed in Vulcan Fast Red chromogen kit (Biocare Medical).

### Statistical analysis

Where required Western blots from three independent experiments were quantified. All statistical analyses were performed using the unpaired, two-tailed Student’s t-test.

## References

[1] Lindblad O (2015). BEX1 acts as a tumor suppressor in acute myeloid leukemia. Oncotarget.

[2] Kazi JU, Agarwal S, Sun J, Bracco E, Rönnstrand L (2014). Src-like-adaptor protein (SLAP) differentially regulates normal and oncogenic c-Kit signaling. J Cell Sci.

[3] Small, D. FLT3 mutations: biology and treatment. *Hematology Am Soc Hematol Educ Program*, 178–184, 10.1182/asheducation-2006.1.178 (2006). 10.1182/asheducation-2006.1.178 17124058

[4] Kabir NN, Rönnstrand L, Kazi JU (2013). FLT3 mutations in patients with childhood acute lymphoblastic leukemia (ALL). Medical oncology.

[5] Kazi JU, Kabir NN, Rönnstrand L (2015). Role of SRC-like adaptor protein (SLAP) in immune and malignant cell signaling. Cell Mol Life Sci.

[6] Puissant A (2014). SYK is a critical regulator of FLT3 in acute myeloid leukemia. Cancer Cell.

[7] Kazi JU, Kabir NN, Flores-Morales A, Rönnstrand L (2014). SOCS proteins in regulation of receptor tyrosine kinase signaling. Cell Mol Life Sci.

[8] Kabir NN, Kazi JU (2014). Grb10 is a dual regulator of receptor tyrosine kinase signaling. Mol Biol Rep.

[9] Moharram SA (2016). Src-like adaptor protein 2 (SLAP2) binds to and inhibits FLT3 signaling. Oncotarget.

[10] Chougule RA (2016). Expression of GADS enhances FLT3-induced mitogenic signaling. Oncotarget.

[11] Zhang J (2015). The Phosphatases STS1 and STS2 Regulate Hematopoietic Stem and Progenitor Cell Fitness. Stem Cell Reports.

[12] Kabir NN, Sun J, Rönnstrand L, Kazi JU (2014). SOCS6 is a selective suppressor of receptor tyrosine kinase signaling. Tumour Biol.

[13] Chougule RA, Kazi JU, Rönnstrand L (2016). FYN expression potentiates FLT3-ITD induced STAT5 signaling in acute myeloid leukemia. Oncotarget.

[14] Kazi JU (2013). The tyrosine kinase CSK associates with FLT3 and c-Kit receptors and regulates downstream signaling. Cell Signal.

[15] Kazi, J. U. *et al*. ABL2 suppresses FLT3-ITD-induced cell proliferation through negative regulation of AKT signaling. *Oncotarget*, 10.18632/oncotarget.14577 (2017). 10.18632/oncotarget.14577 PMC5355336 28086240

[16] Arora D (2011). Protein-tyrosine phosphatase DEP-1 controls receptor tyrosine kinase FLT3 signaling. The Journal of biological chemistry.

[17] Kazi, J. U. *et al*. Tyrosine 842 in the activation loop is required for full transformation by the oncogenic mutant FLT3-ITD. *Cell Mol Life Sci*, 10.1007/s00018-017-2494-0 (2017). 10.1007/s00018-017-2494-0 PMC5487891 28271164

[18] Kazi JU, Rönnstrand L (2013). Suppressor of cytokine signaling 2 (SOCS2) associates with FLT3 and negatively regulates downstream signaling. Mol Oncol.

[19] Kazi JU (2012). Suppressor of cytokine signaling 6 (SOCS6) negatively regulates Flt3 signal transduction through direct binding to phosphorylated tyrosines 591 and 919 of Flt3. The Journal of biological chemistry.

[20] Kazi JU, Rönnstrand L (2012). Src-Like adaptor protein (SLAP) binds to the receptor tyrosine kinase Flt3 and modulates receptor stability and downstream signaling. PLoS One.

[21] Kabir NN, Kazi JU (2011). Comparative analysis of human and bovine protein kinases reveals unique relationship and functional diversity. Genet Mol Biol.

[22] Alarcon B, van Santen HM (2010). Two receptors, two kinases, and T cell lineage determination. Science signaling.

[23] Molina TJ (1992). Profound block in thymocyte development in mice lacking p56lck. Nature.

[24] Majolini MB (1998). Expression of the T-cell-specific tyrosine kinase Lck in normal B-1 cells and in chronic lymphocytic leukemia B cells. Blood.

[25] Koga Y, Kimura N, Minowada J, Mak TW (1988). Expression of the human T-cell-specific tyrosine kinase YT16 (lck) message in leukemic T-cell lines. Cancer research.

[26] Von Knethen A, Abts H, Kube D, Diehl V, Tesch H (1997). Expression of p56lck in B-cell neoplasias. Leukemia & lymphoma.

[27] Seddon B, Legname G, Tomlinson P, Zamoyska R (2000). Long-term survival but impaired homeostatic proliferation of Naive T cells in the absence of p56lck. Science.

[28] Goldsmith MA, Weiss A (1987). Isolation and characterization of a T-lymphocyte somatic mutant with altered signal transduction by the antigen receptor. Proc Natl Acad Sci USA.

[29] Gu TL (2011). Survey of activated FLT3 signaling in leukemia. PLoS One.

[30] Veillette A, Foss FM, Sausville EA, Bolen JB, Rosen N (1987). Expression of the lck tyrosine kinase gene in human colon carcinoma and other non-lymphoid human tumor cell lines. Oncogene research.

[31] Krystal GW, DeBerry CS, Linnekin D, Litz J (1998). Lck associates with and is activated by Kit in a small cell lung cancer cell line: inhibition of SCF-mediated growth by the Src family kinase inhibitor PP1. Cancer research.

[32] Mahabeleshwar GH, Kundu GC (2003). Tyrosine kinase p56lck regulates cell motility and nuclear factor kappaB-mediated secretion of urokinase type plasminogen activator through tyrosine phosphorylation of IkappaBalpha following hypoxia/reoxygenation. The Journal of biological chemistry.

[33] Rouer E, Dreyfus F, Melle J, Benarous R (1994). *Pattern of expression of five alternative transcripts of the* lck gene in different hematopoietic malignancies: correlation of the level of lck messenger RNA I B with the immature phenotype of the malignancy. Cell growth & differentiation: the molecular biology journal of the American Association for Cancer Research.

[34] Leischner H (2012). SRC is a signaling mediator in FLT3-ITD- but not in FLT3-TKD-positive AML. Blood.

[35] Heiss E (2006). Identification of Y589 and Y599 in the juxtamembrane domain of Flt3 as ligand-induced autophosphorylation sites involved in binding of Src family kinases and the protein tyrosine phosphatase SHP2. Blood.

[36] Swords R, Freeman C, Giles F (2012). Targeting the FMS-like tyrosine kinase 3 in acute myeloid leukemia. Leukemia.

[37] Robinson LJ, Xue J, Corey SJ (2005). Src family tyrosine kinases are activated by Flt3 and are involved in the proliferative effects of leukemia-associated Flt3 mutations. Exp Hematol.

[38] Okamoto M (2007). Lyn is an important component of the signal transduction pathway specific to FLT3/ITD and can be a therapeutic target in the treatment of AML with FLT3/ITD. Leukemia.

[39] Lopez S (2016). An essential pathway links FLT3-ITD, HCK and CDK6 in acute myeloid leukemia. Oncotarget.

[40] Casado P (2013). Kinase-substrate enrichment analysis provides insights into the heterogeneity of signaling pathway activation in leukemia cells. Science signaling.

[41] Zhao Y (2015). Identification of potential therapeutic target genes, key miRNAs and mechanisms in acute myeloid leukemia based on bioinformatics analysis. Medical oncology.

[42] Cazzaniga V (2015). LCK over-expression drives STAT5 oncogenic signaling in PAX5 translocated BCP-ALL patients. Oncotarget.

[43] Kazi JU, Sun J, Rönnstrand L (2013). The presence or absence of IL-3 during long-term culture of Flt3-ITD and c-Kit-D816V expressing Ba/F3 cells influences signaling outcome. Exp Hematol.

[44] Razumovskaya E, Masson K, Khan R, Bengtsson S, Rönnstrand L (2009). Oncogenic Flt3 receptors display different specificity and kinetics of autophosphorylation. Exp Hematol.

[45] Blume-Jensen P, Siegbahn A, Stabel S, Heldin CH, Rönnstrand L (1993). Increased Kit/SCF receptor induced mitogenicity but abolished cell motility after inhibition of protein kinase C. EMBO J.

